# Determinants of eating at local and western fast-food venues in an urban Asian population: a mixed methods approach

**DOI:** 10.1186/s12966-017-0515-x

**Published:** 2017-05-25

**Authors:** Nasheen Naidoo, Rob M. van Dam, Sheryl Ng, Chuen Seng Tan, Shiqi Chen, Jia Yi Lim, Mei Fen Chan, Ling Chew, Salome A. Rebello

**Affiliations:** 1 Department of Pathology, Faculty of Medicine and Health Sciences, National Health Laboratory Services, Stellenbosch University, Cape Town, South Africa; 2 Saw Swee Hock School of Public Health, National University of Singapore and National University Health System, Singapore, Singapore; 3 Department of Medicine, Yong Loo Lin School of Medicine, National University of Singapore, and National University Health System Singapore, Singapore, Singapore; 4 Health Promotion Board, Singapore, Singapore

**Keywords:** Eating out, Hawker centres, Fast-food restaurants, Asian population, Mixed methods approach

## Abstract

**Background:**

Like several Southeast Asian countries, Singapore has a complex eating-out environment and a rising eating-out prevalence. However the determinants and drivers of eating-out in urban Asian environments are poorly understood.

**Methods:**

We examined the socio-demographic characteristics of persons who frequently ate away from home in local eateries called hawker centres and Western fast-food restaurants, using data from 1647 Singaporean adults participating in the National Nutrition Survey (NNS) 2010. We also assessed the underlying drivers of eating out and evaluated if these were different for eating at local eateries compared to Western fast-food restaurants using 18 focus group discussions of women (130 women).

**Results:**

Participants reported a high eating-out frequency with 77.3% usually eating either breakfast, lunch or dinner at eateries. Main venues for eating-out included hawker centres (61.1% usually ate at least 1 of 3 daily meals at this venue) and school/workplace canteens (20.4%). A minority of participants (1.9%) reported usually eating at Western fast-food restaurants. Younger participants and those of Chinese and Malay ethnicity compared to Indians were more likely to eat at Western fast-food restaurants. Chinese and employed persons were more likely to eat at hawker centres. The ready availability of a large variety of affordable and appealing foods appeared to be a primary driver of eating out, particularly at hawker centres.

**Conclusions:**

Our findings highlight the growing importance of eating-out in an urban Asian population where local eating venues play a more dominant role compared with Western fast-food chains. Interventions focusing on improving the food quality at venues for eating out are important to improve the diet of urban Asian populations.

**Electronic supplementary material:**

The online version of this article (doi:10.1186/s12966-017-0515-x) contains supplementary material, which is available to authorized users.

## Background

Frequent eating out of the home environment at food retail establishments (‘eating out’) has been shown to be associated with less healthful food choices including lower wholegrain and fruit consumption, higher energy and saturated fatty acid intake and lower intake of micronutrients such as iron, calcium and vitamin C [1, 2]. People who eat out frequently are more likely to gain weight [3] and frequent consumption of Western-style fast-food has been associated with adverse cardio-metabolic outcomes including insulin resistance, type-2 diabetes and heart disease [2, 4, 5]. A growing number of people in several Asian countries eat out frequently, for example, Singapore [6], China [7] and South Korea [8].

This emergent culture of eating-out has been attributed to broader socioeconomic changes including rising affluence, increased participation of women in the workforce [9], urbanization and changes in economic policies allowing for market penetration of transnational food companies and chain restaurants [10]. In addition to Western style fast-food restaurants many Asian, Latin American and African cities have a long tradition of street food vendors and hawkers [11]. Global estimates from the Food and Agricultural Organization suggest that street foods are consumed by 2.5 billion people on a daily basis and they could contribute as much as 40% to daily caloric intake in some Asian cities such as Bangkok [11].

Singapore is an island city-state in Southeast Asia with a diverse population of 3.7 million residents consisting predominantly of Chinese (74%), Malay (13%) and Indian (9%) ethnicities [12]. Singapore has a long tradition of street vendors or hawkers like many of its South-East Asian neighbours [13]. There are more than 100 hawker centres island-wide with each typically housing between 30 and 50 food stalls that provide Singaporeans with a variety of traditional ethnic dishes. These include mixed rice dishes such as fried rice, coconut-rice (“nasi lemak”) and biryani, noodle dishes, stir-fried vegetables, legumes, soy, poultry, meat and fish dishes.

The major Western food chains in Singapore in 2015 were McDonald’s® and Kentucky Fried Chicken® which together accounted for over 55% of the food service value for chained fast-food restaurants [14]. Foods served at these venues are similar to those available internationally but may also include variations to cater to local taste preferences. In light of the rising prevalence of obesity [15] and diabetes [16] in this region, the growth of transnational food chains of Western origin has raised public health concerns [17]. Foods served at these venues are highly palatable, fairly affordable and sophisticatedly marketed but are usually energy-dense and nutrient poor. In Singapore, consumption of Western style fast-foods have been associated with abdominal obesity [2], type-2 diabetes [4] and coronary heart disease [4].

Local vendors seem to provide foods based on a larger variety of food groups, such as legumes and green leafy vegetables, and also employ a diversity of cooking methods including steaming and stir-frying. However, the foods available at these eateries are generally high in sodium and saturated fat and whole grain options are usually limited [18].

The co-existence of local food vendors, Western fast-food outlets and sit-down restaurants gives rise to complex eating-out food environments with multiple types of food providers potentially catering to somewhat different populations. Previous studies, primarily from Western populations, have indicated that eating-out patterns are associated with demographic and socio-economic factors such as age [19–23], ethnicity [19, 24] and occupational status [19]. However, data on the determinants of eating out in an Asian context are sparse.

It is not clear whether the growing number of Western fast-food chains cater to segments of the population with established eating-out patterns or are attracting consumers that may have previously been resistant towards eating at local eateries. This is of interest, given the rising prevalence of eating-out in this region. Also, little is known about the perceptions of consumers towards eating at local versus Western-style venues. In this study of adult Singaporeans we used a concurrent mixed methods approach to examine the socio-demographic characteristics of persons that eat out frequently at Western fast-food restaurants and hawker centres, and used data from focus group discussions to gain an understanding of the food choices made by adult women for themselves and their families to eat out at these venues.

## Methods

We used a concurrent triangulation mixed methods design framework for this study [25]. We used quantitative data from a nationally representative cross-sectional survey to identify the socio-demographic determinants for eating out frequently at Western fast-food restaurants and local eateries. We used qualitative data to understand women’s perceptions of eating at these venues. Both data sources were analyzed separately and results were integrated at the stage of interpretation to understand the determinants of eating out frequently at Western fast-foods and hawker centres in this population with a high eating out prevalence.

### Quantitative survey

We used data from the Singapore National Nutrition Survey (NNS) 2010 for this study. CL and MFC were involved in survey design and in supervising data collection. SC and JYL managed the NNS 2010 survey dataset. The NNS 2010 is a nationally representative survey which monitors population-level food intake and dietary practices. The NNS 2010 comprised of a sub-sample of 1661 of the 4337 individuals who participated in the National Health Survey 2010. Details on the survey methodology of the 2010 National Health Survey have been reported [26]. For the NNS-2010, participants were selected based on a sampling matrix stratified by age, ethnicity and sex with an oversampling of minority ethnicities. To be eligible for the NNS 2010, participants were required to be non-institutionalized Singaporean residents, between 18 and 69 years of age and of either Indian, Malay or Chinese ethnicity. Of the 1661 participants, 14 participants did not meet the age and ethnicity criteria and were excluded from further analyses. The sample size for NNS 2010 was based on detecting a 5% change in energy and macronutrients intake since 2004 (based on the NNS 2004 data) with 90% power and an alpha of 5%. Interviews were conducted by trained interviewers in either English, Malay, Tamil or Mandarin based on participant preference using translated questionnaires. The study was approved by the Singapore Health Promotion Board, Medical and Dental Board Ethics Committee.

The dietary practice questions used in the survey were developed by the Department of Nutrition, Ministry of Health (the current Heath Promotion Board) and have been used in the Singapore NNS since 1998. Access to the survey questionnaire is available upon request to the corresponding author. [6].

This questionnaire consists of 26 multiple-choice or frequency questions including questions about the usual location for consuming meals. Participants were asked about where they usually ate each of the three main meals (breakfast, lunch and dinner). The nine meal location options were: 1) home, 2) packed from home, 3) restaurant/coffee house, 4) workplace/polytechnic/ university canteen, 5) school/college canteen, 6) hawker centre/coffee shop stall/food court, 7) fast-food restaurant, 8) others (to specify), or 9) does not eat the specified meal at all. Intake over a time-frame of over the past week or month were used as probes for participants who had difficulty answering the question. In two separate questions participants were asked how frequently they ate at hawker centres, food courts or coffee shops and how frequently they ate at Western fast-food restaurants (e.g. Kentucky Fried Chicken®, McDonald’s®, Burger King®). Hawker centres are open-air food complexes comprising of multiple food vendors selling prepared local foods. Coffee shop stalls are similar to hawker centres but have a fewer number of stalls (generally under 20). Food courts are air-conditioned hawker centres generally found in larger shopping malls. Since the types of foods sold at these eating venues are fairly similar we will refer to all these eating venues as hawker centres. Demographic information on age, sex, ethnicity, employment status, monthly household income, marital status and highest education were collected using a structured questionnaire.

### Focus group discussions

We conducted a series of focus group discussions in 2011. Inclusion criteria were women of Singaporean Chinese, Malay and Indian ethnicity (*n* = 130) aged between 30 and 55 years. As the three ethnic groups speak different languages, we stratified focus groups on the basis of ethnicity to facilitate group discussions. Since employment status, access to nutrition education and health beliefs can differ by education, we further stratified on the basis of education status (low and high) within each ethnic group. We therefore had 6 focus group discussions per ethnic group (3 groups each for high and low education strata) for a total of 18 focus groups. Group sizes ranged from 5 to 10 participants with a mean of 7 participants per group.

A semi-structured interview protocol was used for the focus group discussions. This guide was developed based on the theory of Triadic Influence [27] which identifies three main influences on health behaviour; biological, social and cultural/environmental. This allowed for development of a priori codes based on these domains. Prompts in the discussion guide related to eating out included questions such as “How do you decide whether to eat at home or to eat out?”, “When and how often do you eat out?” and “What is important to you when selecting food when eating out?” Further details on the study design have been previously published [28]. The study was approved by the Institutional Review Board of the National University of Singapore (reference number: 1330).

### Data analyses

#### Quantitative data analysis

Participants who indicated that they usually obtained meals at non-home locations for two of the three eating occasions, i.e., breakfast, lunch or dinner, were categorized as persons who eat out frequently. We used multivariate logistic regression to assess if frequent eating out was associated with socio-demographic characteristics including age (years), sex (male, female), ethnicity (Indian, Malay, Chinese), marital status (never married, married, divorced or widowed or separated), employment (employed, homemaker, student or national service, retired or unemployed), education (primary or less, secondary, junior college or diploma and degree or profession qualifications) and monthly household income (<$2000 SGD, $2000 - $3999, $4000 - $5999, ≥ $6000). We fitted three models; Model 1 had no adjustments, Model 2 was adjusted for age, sex and ethnicity and Model 3 was further adjusted for education and income to assess if socio-economic and demographic factors in Model 2 (i.e. age, sex and ethnicity) could have independent effects on the outcome. In secondary analyses, we assessed the association between socio-demographic characteristics and eating-out frequently at Western fast-food restaurants and hawker centres. Using the 75th percentile as the cut off, we categorized participants as those who ate 10 or more meals per week at hawker centres as frequent consumers of hawker centre meals and those who ate 1 or more meals per week at Western fast-food restaurants as frequent consumers of fast-foods. *P*-values ≤0.05 were considered as significant. Sampling weights were applied to the data to account for unequal probabilities of selection, nonresponse and oversampling of Malay and Indian ethnicities to better represent national statistics. All analyses were carried out on weighted values of the data using STATA version 11.2.

#### Qualitative data analysis

Audio recorded focus group discussions were transcribed verbatim and then translated into English when necessary. Accuracy of translation was verified by members of the research team who are fluent in Mandarin, Malay, Tamil and ‘Singlish’, a form of English commonly spoken by Singaporeans [29]. Analysis triangulation and thematic coding of the transcripts was done using ATLAS ti (version 6.2.23, ATLAS.ti Scientific Software Development GmbH, Berlin, Germany). Both software aided and manual coding strategies have been previously shown to be advantageous for the qualitative analysis [30]. Coding began with a broad-based reading of all transcript responses with the subsets of questions from the discussion guide initially used to identify broad themes. A thematic analysis approach was used [31] allowing for the flexibility of using a combination of both deductive and inductive approaches in data analysis. The theory of Triadic Influence was used in the initial stages of research for the deductive approach [32] with additional codes added during the course of the analysis thus allowing for the discovery of alternative themes. An inductive approach or ‘bottom up’ approach was used by other members of the research team not involved in the a priori coding. Each researcher independently coded the data and identified themes which were subsequently reviewed within the research team at fortnightly meetings. This allowed for a critical evaluation and consensus of the final themes chosen to best answer the research questions.

## Results

### Participant description and eating-out patterns

Participant demographics were comparable to the overall adult Singapore population [12] in terms of sex distribution, ethnicity and education after weighting (Table 1). However, our participants were older and with fewer never married participants.

**Table 1:** Sociodemographic characteristics of the participants of the Singapore National Nutrition Survey (2010)

	Survey	Survey	Singapore resident population ^a,b^
	N (%)	Weighted %	%
	1647	-	3.7 million
Age (years) ^c^	40 (30–51)	43 (31–54)	37.4
Sex			
Male	817 (49.6)	50.0	49.3
Female	830 (50.4)	50.0	50.7
Ethnicity			
Chinese	673 (40.9)	74.8	74.1
Malay	501 (30.4)	14.5	13.4
Indian	473 (28.7)	10.7	9.2
Others	0 (0.0)	0.0	3.3
Marital status ^d^			
Never married	416 (25.3)	24.7	32.2
Married	1130 (68.7)	69.0	59.4
Widowed/divorced/separated	98 (6.0)	6.3	8.4
Education ^d^			
Primary education or less	277 (16.9)	16.4	22.2
Secondary	591 (36.0)	29.9	29.1
Junior college or diploma	353 (21.4)	19.7	20.1
Degree or professional qualifications	422 (25.7)	34.0	28.6
Employment ^d^			
Employed	1184 (72.0)	73.1	-
Student	89 (5.4)	4.7	-
National service	28 (1.7)	1.4	-
Homemaker/housewife	250 (15.2)	12.4	-
Retired/employed	93 (5.7)	8.4	-
Monthly household income			
< $2000	386 (23.4)	18.3	22.4
$2000 - $3999	489 (29.7)	26.3	18.6
$4000 - $5999	295 (17.9)	16.8	16.4
$6000 and above	311 (18.9)	25.8	42.8
Refused/Don’t know	166 (10.1)	12.8	0.0

^a^Census of population 2010 statistical release 1: demographic characteristics, education, language and religion, Department of Statistics, Ministry of Trade and Industry, Republic of Singapore, Singapore: 2011

^b^Census of population 2010 statistical release 2: households and housing, Department of Statistics, Ministry of Trade and Industry, Republic of Singapore, Singapore: 2011

^c^Values are median (25th,75th percentile)

^d^Due to missing data, numbers for these covariates are as follows: marital status (*n* = 1644), education (*n* = 1643) and employment (*n* = 1644)

**Fig. 1:**
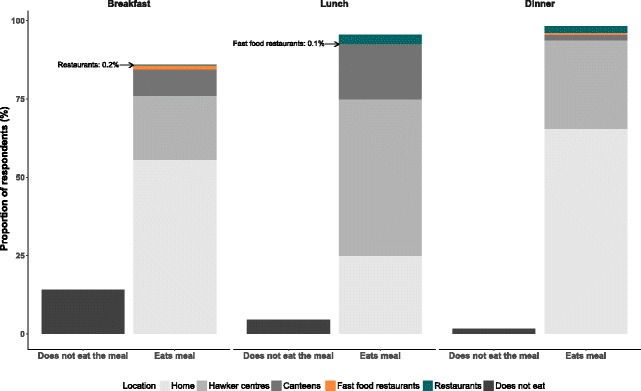
Self-reported venue for usually consuming breakfast, lunch and dinner by participants of the Singapore National Nutrition Survey (2010)

Approximately 77% of participants usually ate out for at least one of the three main meals (breakfast, lunch or dinner) per day with 32.8% usually eating out for 1 meal, 32.6% for 2 meals, 11.9% for all 3 meals **(**Additional file 1: Table S1) and 22.7% did not usually eat out for any of the three main eating occasions. Eating out was most common for lunch, with 70.6% of participants usually eating out for this meal, followed by dinner (32.8%) and breakfast (30.3%) (Fig. 1)**.**

Hawker centres were a popular dining venue with 61.1% of the participants usually eating at least one of the three main meals per day at hawker centres. In contrast only 1.9% of participants usually ate at least 1 meal at fast-food restaurants on a daily basis (Additional file 1: Table S1). Workplace and school canteens were also common dining venues with 20.4% of the participants typically eating at these venue for at least one meal.

### Socio-demographic determinants of eating out

Compared with participants who typically ate out for less than two eating occasions, those that ate out for two or more eating occasions per day were more likely to be male, Chinese and never married (Model 1) (Table 2). Homemakers (spouse who does not work and remains at home to take care of the children and the home) were less likely to eat out frequently compared to working adults. After adjusting for age, sex and ethnicity (Model 2), we observed that participants who were younger, male, of Chinese ethnicity and were employed (compared to homemakers) were more likely to eat out frequently. These results were largely similar after adjusting for education and household income (i.e., Model 3). However, the inverse association between being married and eating out frequently now became statistically significant (Table 2).

**Table 2:** Odds ratio (95% CI) of usually eating-out for 2 or more meals per day by socio-demographic characteristics of participants from the Singapore National Nutrition Survey (2010)^a^

	Usually eat-out for 2 or more meals daily (%)	Model −1^b^	Model -2^b^	Model -3^b^
Eating- out (all locations)^c^	44.5%	-	-	-
Age (years) ^d^	41 (30–54)	0.99 (0.98–1.00)	0.98 (0.97–0.99)*	0.98 (0.96–1.00)
Sex				
Male	51.6	1.00	1.00	1.00
Female	37.4	0.56 (0.41–0.77)*	0.51 (0.36–0.71)*	0.53 (0.37–0.76)*
Ethnicity				
Chinese	52.3	1.00	1.00	1.00
Malay	24.9	0.30 (0.22–0.42)*	0.27 (0.19–0.37)*	0.23 (0.16–0.33)*
Indian	16.9	0.19 (0.13–0.26)*	0.15 (0.11–0.22)*	0.15 (0.10–0.21)*
Marital status				
Never married	54.1	1.00	1.00	1.00
Married	41.4	0.60 (0.43–0.83)*	0.70 (0.46–1.06)	0.60 (0.38–0.96)*
Widowed or divorced or separated	40.9	0.59 (0.30–1.14)	0.96 (0.44–2.08)	1.43 (0.64–3.18)
Education				
Primary or below	38.2	1.00	1.00	1.00
Secondary	46.7	1.41 (0.87–2.30)	1.21 (0.73–2.00)	1.08 (0.62–1.87)
Junior college or diploma	46.9	1.42 (0.85–2.40)	0.85 (0.46–1.57)	0.78 (0.39–1.59)
Degree or professional qualifications	44.3	1.29 (0.75–2.20)	0.72 (0.36–1.43)	0.65 (0.28–1.54)
Monthly household income				
< $2000	42.2	1.00	1.00	1.00
$2000 - $3999	52.6	1.52 (0.98–2.35)	1.25 (0.76–2.07)	1.34 (0.84–2.13)
$4000 - $5999	43.5	1.05 (0.67–1.66)	0.79 (0.46–1.35)	0.91 (0.53–1.55)
$6000 and above	45.6	1.15 (0.73–1.79)	0.74 (0.45–1.21)	0.94 (0.53–1.65)
Employment				
Employed	52.5	1.00	1.00	1.00
Student or national serviceman	40.5	0.62 (0.36–1.05)	0.51 (0.26–1.00)	0.41 (0.18–0.91)*
Homemaker or housewife	8.6	0.08 (0.04–0.18)*	0.11 (0.05–0.24)*	0.07 (0.03–0.17)*
Retired or unemployed	31.2	0.41 (0.15–1.16)	0.37 (0.13–1.07)	0.63 (0.18–2.30)

^a^Estimates are derived from weighted analyses based on sample sizes ranging from 1647 to 1477 due to missing information for specific participant characteristics (household income, *n* = 166;, marital status, *n* = 3;, employment, *n* = 3; and education, *n* = 1)

^b^Model -1: unadjusted; Model -2: adjusted for age, ethnicity and sex; Model -3: further adjusted for education and income

^c^All eating-out locations include hawker centers, food courts, coffee shops, workplace canteens, school, junior college, polytechnic, or university canteens, restaurants or coffee houses, fast-food restaurants and other

^d^Values are median (25th – 75th percentile)

*p-value ≤0.05

We also examined determinants of eating at Western fast-food restaurants and Hawker centres separately. The median frequency (25th, 75th percentile) of eating at hawker centres was 5 times (2, 10) per week whilst the median frequency (25th, 75th percentile) of eating at Western fast-food restaurants was 0 (0, 1) times per week.

We observed no statistical association between age and frequent eating at hawker centres, while participants who were younger were more likely to eat at fast-food restaurants (Table 3). Men were more likely to eat at hawker centres compared to women. Indians and Malays were less likely to eat at hawker centres compared to Chinese. In unadjusted models (Model-1) Malays were more likely to eat at frequently at fast-food restaurants compared to Chinese and Indians. This effect was attenuated after adjusting for age and sex (Model-2). After further adjusting for income and education (Model-3) we observed that Malays were more likely and Indians were less likely to eat frequently at fast-food restaurants as compared to Chinese. Education and income was not statistically associated with frequent eating at hawker centres. While higher educational status was directly associated with frequent eating at fast-food restaurants, this relationship became non-significant in fully adjusted models which included income. As compared to participants with a monthly household income of under 2000 SGD, those with an income of 4000 SGD and above were more likely to eat frequently at fast- food restaurants. This association was attenuated after adjusting for covariates. Employed persons were more likely to eat at hawker centres compared to homemakers or students. Employed persons were more likely to eat frequently at fast-food restaurants compared to homemakers, but less likely to do so compared to students. These associations became statistically non-significant in adjusted models.

**Table 3:** Odds ratio (95% CI) of eating frequently at hawker centers (10 or more meals per week) or fast-food restaurants (1 or more meals per week) by socio-demographic characteristics of participants from the Singapore National Nutrition Survey (2010)^a^

	Hawker centers 10 or more meals per week (27.1%)			Western fast-food restaurants 1 or more meals per week (27.3%)		
	Model 1^b^	Model 2^b^	Model 3^b^	Model 1^b^	Model 2^b^	Model 3^b^
Age (years)	1.01 (1.00–1.02)	1.00 (0.99–1.02)	1.01 (0.99–1.03)	0.92 (0.90–0.94)*	0.92 (0.90–0.94)*	0.93 (0.91–0.95)*
Sex						
Male	1.00	1.00	1.00	1.00	1.00	1.00
Female	0.57 (0.39–0.84)*	0.54 (0.36–0.81)*	0.64 (0.42–0.96)*	0.78 (0.56–1.09)	0.66 (0.46–0.96)*	0.71 (0.49–1.03)
Ethnicity						
Chinese	1.00	1.00	1.00	1.00	1.00	1.00
Malay	0.16 (0.10–0.24)*	0.16 (0.10–0.24)*	0.12 (0.08–0.20)*	1.68 (1.22–2.30)*	1.31 (0.93–1.84)	1.44 (1.00–2.06)*
Indian	0.15 (0.10–0.24)*	0.15 (0.10–0.23)*	0.13 (0.08–0.21)*	0.76 (0.54–1.08)	0.50 (0.34–0.74)*	0.50 (0.33–0.74)*
Marital status						
Never married	1.00	1.00	1.00	1.00	1.00	1.00
Married	1.15 (0.78–1.68)	1.03 (0.64–1.65)	1.08 (0.66–1.79)	0.31 (0.22–0.45)*	1.32 (0.84–2.07)	1.21 (0.73–2.02)
Widowed/divorced/separated	0.92 (0.44–1.93)	0.94 (0.37–2.39)	1.33 (0.50–3.50)	0.11 (0.05–0.25)*	0.83 (0.30–2.33)	0.81 (0.27–2.40)
Education						
Primary or lower	1.00	1.00	1.00	1.00	1.00	1.00
Secondary	1.15 (0.62–2.14)	1.26 (0.66–2.39)	1.02 (0.51–2.05)	4.02 (2.18–7.38)*	1.99 (1.04–3.80)*	1.62 (0.81–3.23)
Junior college or diploma	1.01 (0.52–1.94)	1.00 (0.46–2.18)	0.84 (0.36–1.96)	11.18 (5.97–20.93)*	2.94 (1.40–6.15)*	2.07 (0.94–4.54)
Degree or professional qualifications	1.12 (0.57–2.20)	0.97 (0.42–2.23)	0.83 (0.31–2.19)	6.63 (3.45–12.76)*	2.47 (1.06–5.77)*	1.74 (0.71–4.26)
Monthly household income						
< $2000	1.00	1.00	1.00	1.00	1.00	1.00
$2000 - $3999	1.56 (0.94–2.56)	1.43 (0.79–2.59)	1.48 (0.84–2.60)	1.45 (0.92–2.28)	1.24 (0.78–1.98)	1.19 (0.74–1.92)
$4000 - $5999	1.17 (0.66–2.07)	1.02 (0.54–1.93)	1.10 (0.59–2.03)	1.66 (1.05–2.64)*	1.38 (0.84–2.26)	1.25 (0.74–2.13)
$6000 and above	1.03 (0.58–1.80)	0.75 (0.41–1.37)	0.83 (0.44–1.58)	1.72 (1.03–2.85)*	1.51 (0.82–2.80)	1.34 (0.75–2.41)
Employment						
Employed	1.00	1.00	1.00	1.00	1.00	1.00
Student/national serviceman	0.18 (0.08–0.39)*	0.20 (0.09–0.48)*	0.17 (0.06–0.49)*	4.48 (2.51–8.00)*	0.86 (0.45–1.66)	0.73 (0.34–1.59)
Homemaker/housewife	0.11 (0.05–0.25)*	0.12 (0.05–0.32)*	0.10 (0.03–0.31)*	0.31 (0.18–0.53)*	0.74 (0.40–1.37)	0.94 (0.49–1.80)
Retired/unemployed	0.72 (0.23–2.31)	0.53 (0.15–1.81)	0.83 (0.18–3.75)	0.71 (0.19–2.71)	2.43 (0.50–11.77)	3.26 (0.66–16.21)

^a^Estimates are derived from weighted analyses based on sample sizes ranging from 1647 to 1477 due to missing information for specific participant characteristics (household income, *n* = 166; marital status, *n* = 3; employment, *n* = 3 and education, *n* = 1)

^b^Model 1: unadjusted; Model 2: adjusted for age, ethnicity and sex; Model 3: further adjusted for education and income

*p-value ≤0.05

### Focus group discussions on eating at hawker centres and Western fast-food restaurants

Four themes consistently emerged in relation to perceptions of hawker stalls in Singapore. These were large variety, convenience, unhealthy food and lack of cleanliness. The design of hawker centres which provides consumers with a wide variety of cuisine styles under one roof was often cited as a reason for choosing to eat at hawker centres.

*“I mean in the hawker centres, we have more varieties. It is a bigger area, and there are a lot of stalls.”* (Chinese, lower education).

Only a few participants framed this diversity of choices in the context of healthfulness of dishes, with some women indicating that both healthful and less healthful options are available and others were unsure if this was the case.

*“A lot of choice [at hawker centres] but I can’t be sure whether it is healthy or unhealthy.”* (Malay, lower education).

Convenience with respect to economic convenience in terms of low cost and physical convenience in terms of ease of access was cited as reasons for eating at hawker centres. Some women highlighted the convenience of eating out in relation to busy work schedules and lack of time. Home cooking was described as being hard to achieve whilst working but was more feasible once women stopped working.

“*…now that I’m still adjusting to the working (life), we find that it’s so much more convenient to eat out and usually it’s the children (who) would like to eat something which is different. So they would want Western, not the Malay rice and…but I don’t like us to eat as a family…to eat at fast-food restaurants because it’s…to me you can get something more substantial at a cheaper and at a lower price (at a hawker centre).” (Malay, higher education).*

*“They are all working. The food outside is easy to get. I do (did) not know how to cook, but now I do because I am no longer working. But if I was working and food is easy to get, outside, I will be less skilled.”* (Malay lower education).

Hawker centre foods were often described as unhealthy because of the amount or quality of the ingredients such as the use of monosodium glutamate and less healthful cooking oils or practices such as recycling oil.

*“…what gives me the impression (that the hawker centre food) is not healthy, because I don’t see a lot of veggie(s) or fruits, which doesn’t tell (say) much, and tells us on top of that, they are all fried and (hawker) food centre(s) normally tend to recycle their cooking oil.”* (Chinese lower education).

Some respondents discussed strategies to eat healthfully at hawker centres such as sharing a meal, choosing vegetables and steamed options and buying foods from specific stalls that serve more healthful meals. Compared to restaurants, hawker centres were perceived by some participants to be less clean, described as being hot, smoky, having inadequate housekeeping and a possibility of falling ill because of poor hygiene practices.

The perceived unhealthfulness, convenience and variety of Western fast-foods and its popularity amongst youth emerged as major themes across the different focus groups. Western fast-foods were generally regarded as being fatty, processed, energy dense and described as being unhealthy.

*“…the food is less healthy but in time of need, it is needed too. [laughter]. If we go anywhere where there isn’t halal food, we are forced to eat it (Western fast-foods) because that (is the) case, at my house.”* (translated from Malay; Malay higher education).

The attractiveness of fast-foods to children and youth emerged as a salient theme. This was attributed to the appealing taste of these foods, more autonomous decision making in children due to the availability of pocket money and the use of these foods as comfort foods given stressful school schedules.

*Respondent: “Because they like junk food very much. They are given money they are always thinking of going (to) McDonalds and KFC. They enjoy eating I don’t know whether (they) know about it (potential health effects of frequent eating at fast-food restaurants). Even if they know the impact, they don’t care because they enjoy it.. they love it. Whenever, I want to give my students a treat, they always say “teacher, KFC”. I say it’s not healthy.. once in a while it’s okay.. I’ve some students who must drink cold [soft] drinks, they said “teacher, after eating my lunch I must have a glass of that”. They are hooked on to that.” (Indian higher education).*

Some women discussed strategies to reduce the intake of unhealthy foods amongst children including eating at home before going out and highlighting the role of schools in improving children’s dietary intakes. Western fast-foods were described as being convenient because of their easy accessibility, late operating hours, and the availability of home-delivery option which is a service not provided by hawker centres. These foods were seen by some women as contributing to the culinary diversity of food choices particularly favoured by their children.

*“No, my children like varieties, something like western food. But I only cook Indian food, so only sometimes we argue. For us we’ll (ask) our children what type of food to cook (and they reply) “Ah ya, you always cook curry curry curry, told you not to cook curry already. You don’t know how to cook other” pasta, lasagna"… they like that type (of food). But I don’t know how to cook (it)! I say, “I’m very sorry.”…(the children reply) “We want to go outside and eat.” (Indian lower education).*

## Discussion

Using data from the nationally representative Singapore National Nutrition Survey 2010 and focus group discussions of 130 Singaporean women, we explored participants’ perceptions of eating out at Western and traditional Asian venues to provide insights into reasons why people eat at these venues. We also identified the socio-demographic attributes that characterize persons who eat out frequently at these venues. We observed that eating out frequently was more common amongst men, employed, compared to home-makers, and those of Chinese ethnicity. Persons who ate out frequently at hawker centres were more likely to be of Chinese ethnicity whilst those eating out frequently at Western fast-food restaurants were more likely to be of Malay or Chinese ethnicity. Variety, convenience and unhealthy foods were themes that emerged in focus group discussions that centred on eating at hawker centres or Western fast-food restaurants. Hawker centres were perceived by some as being venues that were less sanitary whilst Western fast-food restaurants were thought to be particularly appealing to youth.

Findings from our study highlights the predominant eating-out culture in Singapore with 77.3% of our participants regularly obtaining at least one main meal out of home daily. This is supported by household expenditure survey data which indicate that on average Singaporeans spend more than 60% of their food expenses on away from home food [33]. In comparison, eating out constitutes 41% of the food budget in the US [34] and 21% in urban China [35]. The high eating-out prevalence observed in our study could be explained partly by both the remarkably rapid socio-economic changes Singapore has experienced over the past few decades [36] and due to rental policies that have historically subsidized eating out at hawker centres making this a convenient and economical alternative to home-cooking [37].

Since independence in 1965, Singapore has undergone transformative economic reforms and now has one of the lowest unemployment rates and one of the highest GDP per capita globally [36, 38]. Results from both our survey and focus group discussion highlight the importance of employment as a determinant of eating out. Consistent with data from other Western populations, we observed that participants who were employed had a higher likelihood of eating out more frequently [19, 39]. Lack of time for food preparation and greater disposable income has been cited as a possible reasons for the greater reliance on outside foods in employed persons [40]. Women in our focus groups also highlighted the convenience of eating out as a way to better manage perceived time scarcity due to work demands.

The role of income in eating out frequency may vary based on whether this is viewed as a luxury or a necessity. Compared to other foods, price-elasticity studies suggest that dining out is considered more of a luxury [41–43] although this may be less valid for urban single adults [43]. In line with this, frequency of consumption of food away from home in US working age adults decreased from 2005 to 2010, a period that was characterized by high unemployment rates and increasing food prices [40]. However, data from other studies suggest that the differences in time spent on cooking by employment status are modest and income decreases may result in changes in the acquisition of food from less economical eating venues such as sit-down restaurants to more economical venues such as fast-food restaurants [44]. Other aspects of employment such as the increased opportunity to access away-from home eating venues may also be important drivers of eating out and indeed we noted workplace and school cafeterias as a very popular eating-out venue. In the Singapore context, eating at local hawker centres is inexpensive (a meal typically costing 3–4 USD) and was regarded as such by our focus group participants. Perceived scarcity of time, rather than disposable income, resonated more strongly with the women in our focus group discussion. Disposable income in the form of pocket money may be a more relevant driver of eating out behaviour in children and teens who appear to favour the more expensive Western fast-food restaurants [45]. This is consistent with our focus group findings where women perceived that the availability of pocket money enables children to eat out at Western fast-food chains.

We observed considerably higher eating-out frequencies in Chinese compared to Malay and Indian participants. These ethnic differences were not explained by variability in income or education and may be related to cultural or environmental factors. Traditional expectations of the woman’s role in cooking may persist more in some ethnic communities compared to others [46]. Also, the efficiency of eating out with less perceived time-cost and food wastage may resonate more strongly amongst Chinese with deeply held Confucian values of productivity, while Indians may have a preference for home-cooked meals for health reasons [46]. Data from our focus group discussion suggests that the limited number of halal certified hawker stalls may prevent Malays, who typically follow Islamic traditions, to eat out more frequently. Malay adults were more likely to eat at fast-food restaurants, which tend to be halal-certified, compared to Chinese or Indians. This suggests that whilst the lack of convenient eating out venues may motivate Malay participants to eat home cooked food such that when they do eat out it is likely to be at venues such as fast-food restaurants. The role of environmental factors in contributing to ethnic disparities with regards to food access is increasingly being recognized in Western countries [47] and needs to be investigated in Asian food environments.

Contrary to the US where Western fast-foods account for nearly 40% of total calories consumed away from home [48], local eating venues in Singapore are the primary source of away from home prepared foods. Market research data suggest that this may be the case for other Asian countries as well [14]. In 2014, Asian fast-food eateries comprised 96.6% of the 1.53 million fast-food outlets in China, 94.7% of the 87,186 outlets in India and 68.6% of 8152 outlets in Vietnam [14]. Unlicensed street food vendors also form an important part of the food distribution networks in many South East Asian countries [11].

Additionally the impetus for selecting Western fast-foods appears to differ in Asian and Western contexts. Although convenience and low cost are some of the major reported drivers for Western style fast-food consumption in the US [49] and Australia [50], our data suggest that these factors may not be of singular importance in Singapore. In our focus group discussions, convenience and value for money was more frequently reported as themes related to hawker foods, rather than Western fast-foods. With low overhead costs due to lack of air-conditioning and wait-staff, hawkers are able to offer cooked foods at lower prices compared to other eating venues. Also, the rental leases of many hawkers are subsidized by the government [37]. These rental subsidies can be traced back to the 1970’s when the government relocated mobile street vendors to newly constructed hawker centres which were strategically located near residential neighbourhoods and business centres as a way to regulate the quality of foods sold [37]. Apart from their easy accessibility, the large variety of dishes available at these hawker centres also emerged as a salient theme in our focus group discussions. This allows for families to cater to the diverse food preferences of various family members [28, 46].

Other studies suggest that Western fast-foods have more of a symbolic or social value and less of a utilitarian function in Asian cultures [8, 9, 51]. Young Indian adults viewed eating at fast-food restaurants as fun and entertaining but did not consider this as a substitute for home foods [51]. Similar recreational attributes to Western fast-foods have been made by Filipino, Malay and Indonesian adults [9]. In our study, Western fast-foods was recognized as adding variety to usual food experiences. Concerns about food safety and hygiene practices at hawker centres were frequently discussed and this may lead to eating at alternate eating venues, including Western fast-food restaurants.

The popularity of fast-foods amongst children and youth emerged as a salient theme in most groups. Consistent with this we observed that younger persons were more likely to eat at Western fast-foods restaurants. Although data in Singaporean children and teens are lacking, we observed that 70.8% of adults aged 18–21 years consumed Western fast-foods on a weekly basis compared to 3.0% of adult aged 60 years and over. High consumption of these foods in the younger age demographic has also been observed in studies from US [19, 21, 22], Europe [20] and South Korea [23]. In Singapore, children play a key role in family food decisions [46]. Accommodating their food preferences is seen as being efficient and less wasteful [46]. Children have been reported to exert considerable ‘pester-power’ in influencing food shopping decisions made at supermarkets in the US [52]. Parents from India, Malaysia and Pakistan also reported children as being key influencers in choosing food products [53].

The perceived unhealthfulness of hawker-centre foods were related to vendor practices such as recycling oils, poor sanitary standards, and lack of vegetables. Although vegetable dishes are available at most hawker-centres this may vary by stall, with some stalls serving cuisines such as *yong tau foo* providing a greater variety of vegetables, and others such as *chicken rice* providing limited vegetable options.

The use of a mixed methods approach to characterize and examine determinants of eating out behaviours in a multi-ethnic Asian setting with a large nationally representative sample is a strength of this study. However, like most studies examining dietary behaviours, our results are based on self-reported eating out behaviours and reporting errors are possible. Our focus group discussion groups consisted of women and did not include men, who were most likely to eat out frequently. However, this provided us with insights on why women, who are typically regarded as the gatekeepers of family food decisions, choose to eat out [46]. Future studies could focus on snacking patterns, which were not captured by our survey, and on perceptions and drivers of eating out in men, children and teens.

## Conclusions

Taken together, our data suggest that concerns about the healthfulness and hygiene of foods sold away from home are out-weighed by their convenience and affordability. Faced with multiple demands on their time and attention, women viewed eating out as efficient. Men, younger, and employed persons may be particularly affected by the drivers of eating out and children may initiate the family’s decisions to eat at Western fast-food restaurants. The Singapore experience suggests that as Asian cities undergo a socio-economic transition, regular home cooking may increasingly become less attractive and eating out more common. Local food retailers such as hawkers are important food providers and must be considered in policies that seek to address the nutrition transition and rising rates of cardio-metabolic diseases in this region [16, 54]. In Singapore, the Health Promotion Board has initiated the Healthier Dining Program to make healthier options more available at external eating venues [55]. This initiative and possibly initiatives that promote home-cooking may also be relevant to other Asian settings such as Malaysia and Hong Kong with similar food establishment models.

## Additional file

Additional file 1: Frequency (percent) of eating out at different locations for daily meals in participants of the Singapore National Nutrition Survey (2010). (DOCX 14 kb)

## Abbreviations

NNS: National Nutrition Survey
SGD: Singapore Dollar
USD: United States Dollar
