# Supplementary material for: Determinants of eating at local and western fast-food venues in an urban Asian population: a mixed methods approach
Source: Int J Behav Nutr Phys Act. 2017 May 25;14:69. doi: 10.1186/s12966-017-0515-x (PMC5445485; doi:10.1186/s12966-017-0515-x)
Supplement: Additional file 1: — Frequency (percent) of eating out at different locations for daily meals in participants of the Singapore National Nutrition Survey (2010). (DOCX 14 kb) [file 12966_2017_515_MOESM1_ESM.docx]

| **Additional file 1: Table S1.** Frequency (percent) of eating out at different locations for daily meals in participants of the Singapore National Nutrition Survey (2010) ^1,2^ | | | | |
| --- | --- | --- | --- | --- |
| Different locations | Not usually | Usually 1 meal | Usually 2 meals | Usually 3 meals |
|  |  |  |  |  |
| Home or packed from home | 19.2 | 33.1 | 30.3 | 17.4 |
| All non-home locations | 22.7 | 32.8 | 32.6 | 11.9 |
| Restaurants | 95.6 | 3.5 | 0.8 | 0.1 |
| Workplace and school canteens | 79.6 | 14.0 | 5.5 | 0.9 |
| Hawker center | 38.9 | 30.5 | 23.6 | 7.0 |
| Fast food restaurants and others | 98.1 | 1.8 | 0.1 | 0.0 |

The nine meal location options were re-categorized into 5 broad groups based on similarity of the eating establishments: 1) home or packed from home 2) restaurant/coffee houses 3) workplace/polytechnic/university canteens and school/college canteens 4) hawker centre/coffee shop stall/food court and 5) fast food restaurant/others.

Row percents add up to 100%
